# Exosomal Circular RNA hsa_circ_0046060 of Umbilical Cord Mesenchymal Stromal Cell Ameliorates Glucose Metabolism and Insulin Resistance in Gestational Diabetes Mellitus via the miR-338-3p/G6PC2 Axis

**DOI:** 10.1155/2022/9218113

**Published:** 2022-06-11

**Authors:** Minkai Cao, Chaozhi Bu, Jingjing Zhang, Yongwei Ren, Guanlun Zhou, Chao Chen, Guorong Han, Shi-Wen Jiang, Juan Wen

**Affiliations:** ^1^Department of Gynecology and Obstetrics, The Second Hospital of Nanjing, Nanjing University of Chinese Medicine, Nanjing 210003, China; ^2^Department of Obstetrics and Gynecology, The Affiliated Wuxi Matemity and Child Health Care Hospital of Nanjing Medical University, Wuxi 214002, Jiangsu, China; ^3^Center of Reproductive Medicine, State Key Laboratory of Reproductive Medicine, Research Institute for Reproductive Health and Genetic Diseases, The Affiliated Wuxi Matemity and Child Health Care Hospital of Nanjing Medical University, Wuxi 214002, Jiangsu, China; ^4^Department of Obstetrics and Gynecology, Qilu Hospital of Shandong University, Jinan 250012, Shandong, China; ^5^Nanjing Maternity and Child Health Care Institute, Women's Hospital of Nanjing Medical University, Nanjing Maternity and Child Health Care Hospital, Nanjing 210004, China

## Abstract

**Background:**

Impaired glucose metabolism and insulin sensitivity have been linked to the pathogenesis of gestational diabetes mellitus (GDM). Exosomes secreted by the umbilical cord mesenchymal stromal cells (UMSCs) and circular RNAs (circRNAs) derived from exosomes have been shown to be associated with the progression of GDM-related complications.

**Methods:**

UMSCs were isolated from umbilical cords and identified through flow cytometry. Exosomes were isolated from UMSCs and were then characterized. The expression levels of RNA of hsa_circ_0046060, mmu_circ_0002819, and miR-338-3p were determined by quantitative real-time polymerase chain reaction (RT-qPCR). The intracellular glucose intake and glycogen content were measured using a High Sensitivity Glucose Assay Kit and Glycogen Assay Kit, respectively. Bioinformatics analysis and luciferase reporter assay were used to validate interactions among hsa_circ_0046060, miR-338-3p, and G6PC2. The expression of insulin receptor substrate-1 (IRS-1) and its phosphorylated form, (p-IRS-1), as well as G6PC2, was determined through western blotting.

**Results:**

UMSCs and exosomes were successfully isolated and identified. The upregulation of hsa_circ_0046060 decreased the intracellular glucose content in L-02 cells (43.45 vs. 16.87 pM/mg, *P*=0.0002), whereas shRNA-mediated downregulation reversed this effect (16.87 vs. 33.16 pM/mg, *P*=0.0011). Mmu_circ_0002819 in mice aggravated dysregulated glucose metabolism (49.88 vs. 21.69 pM/mg, *P*=0.0031) and insulin sensitivity (0.20 vs. 0.11 mg/mL, *P*=0.03) in GDM mice, which was abrogated by the knockdown of mmu_circ_0002819. The results of luciferase reporter assay revealed that miR-338-3p and G6PC2 were the potential targets of has_circ_0046060. Western blotting results showed that the reduced activation of IRS-1 induced by GDM (1.25 vs. 0.54, *P*=0.0001) could be rescued by the administration of si-circ-G-UMSC-EXOs (0.54 vs. 1.17, *P*=0.0001).

**Conclusion:**

Taken together, the inhibition of hsa_circ_0046060 expression in exosomes from GDM-derived UMSCs can alleviate GDM by reversing abnormal glucose metabolism and insulin resistance *in vivo* and *in vitro*.

## 1. Introduction

Increased insulin resistance (IR) and immune tolerance in pregnant women result in metabolic and immunological alterations, which may aggravate the development of gestational diabetes mellitus (GDM). GDM characterized by irregular glucose intolerance was initially reported during pregnancy [1]. GDM is associated with the increased risk of several maternal and neonatal complications, including caesarean section, giant infants, premature delivery, stillbirths, and neonatal hypoglycemia, and it may advance to type 2 diabetes after delivery within 10–15 years [2–4]. Although the precise pathogenic mechanism of GDM has not been fully elucidated, it may occur as a result of abnormal glucose regulation and increased IR, inducing the disorders of glucose metabolism. Studies report that circular RNAs (circRNAs) derived from exosomes may play a key role in GDM development.

CircRNAs are newly discovered noncoding RNAs formed through the alternative splicing of premessenger RNA (mRNA). They have a characteristic closed-loop structure with connected 3′ and 5′ ends [5, 6]. CircRNAs are more resistant to degradation compared with mRNAs and exhibit high biological stability because of the complete circular covalently linked structure [7, 8]. High-throughput RNA sequencing and bioinformatics analysis have led to the identification of several novel circRNAs that exhibit biological characteristics and regulatory functions [6, 9]. Previous findings indicate that circRNAs mainly function as microRNA (miRNA) sponges to regulate the transcription and posttranscription of miRNA-targeted genes. As a result, circRNAs exert important roles in numerous physiological and pathological processes [9, 10]. The dysregulation of the expression of circRNAs is implicated in the occurrence and progression of several diseases, including preeclampsia and GDM [11–14].

Exosomes are small extracellular vesicles (EVs), 30–150 nm in diameter. Exosomes contain multiple biomolecules, including glycans, nucleic acids, and proteins. They transfer biologically-active materials for intercellular communication between cells and are the vital modulators of physiological and pathological status [15]. Exosomes act as potential effectors in regulating the biological functions of umbilical and placental cells involved in the pathogenic processes of pregnancy complications, including GDM and preeclampsia [11, 12, 16]. Intact and stable circRNAs have been identified in exosomes [17, 18]. However, the potential mechanism of circRNAs on GDM has not been fully elucidated.

The mesenchymal stromal cell (MSC) is a class of adult stem cell family. They were initially identified in the bone marrow but have been recently reported in other tissue or organs, including peripheral blood and umbilical cord blood (UCB) [19]. Human umbilical cord mesenchymal stromal cells (hUMSCs) are characterized by low cost, superior viability, versatility, and low immunogenicity. Thus, they are more effective and suitable for tissue repair and replacement therapy [20, 21]. Noncoding RNAs, including miRNA and circRNA in exosomes derived from hUMSCs (hUMSC-EXOs), are implicated in the modulation of several biological processes, such as cell proliferation, pyroptosis, and differentiation, and they exhibit neuroprotective activities [22, 23].

A microarray assay was previously conducted for comparative circRNA profiling of umbilical cord blood exosomes derived from GDM patients and matched controls for maternal age and gestational age [24]. The microarray data showed the aberrant expression of several circRNAs, in which hsa_circ_0046060 expression was upregulated in GDM patients. Therefore, hsa_circ_0046060 was selected for subsequent functional experiments to explore its regulatory effects on the pathogenesis of GDM. In the present study, the results showed that hMUSC-derived exosomal hsa_circ_0046060 affected the glucose uptake and IR of normal human liver cell L-02 and GDM mice by modulating the miR-338-3p/G6PC2 axis. These findings provide a better understanding of the functional roles of hsa_circ_0046060 in the pathogenesis of GDM. In addition, circ_0074673 is a potential biomarker and therapeutic target for GDM.

## 2. Materials and Methods

### 2.1. Ethics Statement

Patient enrollment and sampling processes were done as stipulated by the Declaration of China and relevant local regulatory guidelines. Prior to inclusion, all patients were required to provide a written informed consent, and their privacies were strictly protected. Animal procedures were conducted with reference to the Institutional Animal Ethical Committee of the Affiliated Wuxi Maternity and Child Health Care Hospital of Nanjing Medical University guidelines. The study performed using the clinical samples and animal procedures was approved by the Ethics Committee of the Affiliated Wuxi Maternity and Child Health Care Hospital of Nanjing Medical University (No. 2020-01-0824-32).

### 2.2. Cell Culture and Establishment of Insulin Resistance Cell Model

The human liver cell line L-02 was cultured in 10% FBS-supplemented RPMI-1640 medium (Wisent) along with 100 U/mL penicillin and 100 *μ*g/mL streptomycin at 37°C in an environment of 95% humidity and 5% CO_2_. The incubation of L-02 cells for 24 h was done in the presence of palmitic acid (Sigma-Aldrich) to build an insulin-resistant cell model.

### 2.3. Isolation, Characterization, and Culture of UMSCs

The GDM patients were pregnant women whose 2 h plasma glucose was ≥8.0 mmol/L or fasting blood glucose was ≥5.5 mmol/L after a 75 g oral glucose tolerance test (OGTT). The establishment methods of GDM mouse model were described in the subsequent section. hUMSCs were isolated from umbilical cords' blood that were freshly collected from patients and healthy donors with caesarean delivery and identified, and mUMSCs were isolated from the umbilical cord of control and GDM mice on gestational day (GD) 20 using similar protocols to human. UMSCs were cultured in 10% FBS (Gibco, USA)-supplemented low-glucose Dulbecco's modified Eagle's medium (L-DMEM, Wisent) and 100 U/mL of streptomycin/penicillin in a 5% CO_2_ atmosphere at 37°C, and they were used for experiments in 3 to 5 passages. UMSCs were stained with FITC-conjugated antibodies against CD34, CD45, CD90, and CD105 (eBioscience) in ice for 20 min away from light, and cells were analyzed by flow cytometry (BD).

### 2.4. Isolation, Characterization, and Treatment of Exosomes

The cell culture supernatants of UMSCs containing exosomes were centrifuged for 20 min at 2000 *g* and 4°C, followed by 30 min of centrifugation at 10, 000 *g* and 4°C. The filtration of the supernatants was done via a 0.22 *μ*m filter (Millipore). Exosomes were isolated from the filtered supernatant via the total exosome isolation kit (Ribobio), as instructed by the manufacturer. Protein concentrations of isolated exosomes were determined by a BCA protein assay kit (Beyotime). Final UMSC-derived exosomes (UMSC-EXOs) concentrations for *in vitro* assays was 400 *μ*g/mL and 10 mg/kg for *in vivo* assays. UMSC-EXOs morphologies were observed by transmission electron microscopy (JEM1011). Exosomal protein markers (CD63 and CD81) were evaluated by western blotting. Size distributions of exosomes were assessed by nanosight tracking analysis (NTA, nanosight). In our study, we obtained differentially sourced exosomes, inducing exosomes derived from the cultured UMSCs of GDM (G-UMSC-EXOs), exosomes derived from the cultured UMSCs of controls (C-UMSC-EXOs), and si-cirRNA-treated exosomes derived from the cultured UMSCs of GDM (si-circ-G-UMSC-EXOs).

### 2.5. RNA-Fluorescence In Situ Hybridization (RNA FISH)

This assay was done using the fluorescent in situ hybridization kit (Ribobio) as instructed by the manufacturer. Firstly, L-02 cells were washed twice using phosphatebuffered saline (PBS) and thereafter fixed for 10 min in formaldehyde (4%). Triton X-100 was used to permeabilize the fixed cells for 5 min. Permeabilized cells were washed twice using PBS, after which they were incubated overnight away from light in the presence of Cy3-labeled probes specific to the hsa_circ_0046060 back-splice region at 37°C. After the addition of the DAPI working solution, the Zeiss LSM710 confocal fluorescence microscope was used to scan and image the cells.

### 2.6. Exosome Labeling

The red fluorescent membrane dye PKH67 (Sigma) was used to label C-UMSC-EXOs and G-UMSC-EXOs. L-02 cells were grown in 6-well plates to 80% confluence. Then, the medium was replaced with RPMI-1640 medium with PKH26-labeled exosomes. After 24 h of incubation at 37°C in a 5% CO_2_ environment, cells were washed twice using PBS and fixed, after which their nuclei were DAPI stained and observed by fluorescence microscopy (Nikon).

### 2.7. RNase *R* Treatment

The incubation of total RNA (2 *μ*g) extracts from L-02 cells was done for 30 min in the presence of RNase *R* (Epicentre, 8 U) at 37°C. Then, RNA was subjected to RT-qPCR assays to assess the stability of hsa_circ_0046060.

### 2.8. Cell Transfection

The L-02 cells were seeded in 6-well plates followed by incubation for 24 h until they reached 60–70% confluence. Then, they were transfected with small-interfering RNAs (siRNAs, Genepharma), targeting hsa_circ_0046060, mmu_circ_0002819, hsa-miR-338-3p, and G6PC2. Transfection was done using the lipofectamine 3000 reagent (Invitrogen), as instructed by the manufacturer. After 4–6 h of incubation, L-02 cells were recovered with fresh medium and at 24–48 h post-transfection, cells were obtained for further assays. siRNA knockdown efficiencies were evaluated by qRT-PCR. Supplementary Table S1 show the siRNA sequences.

### 2.9. Knockdown of hsa_circ_0046060 and mmu_circ_0002819 via Lentiviral Vector Transduction

The transduction of UMSCs at 200 particles/cell multiplicity of infection was performed using Lentiviruses expressing hsa_circ_0046060 and mmu_circ_0002819 inhibitors. These assays were conducted in 24-well plates in RPMI-1640 medium, incubated at 37°C in a 5% CO_2_ environment for three days. RT-PCR was performed to assess UMSC and hUMSC-EXOs hsa_circ_0046060 and mmu_circ_0002819 levels to confirm successful transduction. The control used in this assay was the no-load shRNA lentivirus.

### 2.10. Luciferase Assay

Full lengths of wild-type and mutant 3′ UTR of hsa_circ_0046060 and G6PC2mRNA were amplified by PCR and inserted into pGL6-miR-Report double luciferase report vectors (Generalbiol). Then, Lipofectamine 3000 (Invitrogen) was used to transfect the cells with luciferase report vectors and 200 nM miR-338-3p (5′-UCCAGCAUCAGUGAUUUUGUUG-3′) mimic and negative control (NC)-miR-338-3p (5′-UUCUCCGAACGUGUCACGUTT-3′). Luciferase activities were evaluated after 48 h via the dual-luciferase reporter assay system (Promega, USA) as instructed by the manufacturer. Normalized luciferase activities were evaluated as relative values of Firefy luciferase activities to Renilla luciferase activities.

### 2.11. Development and Treatment of GDM Mice Models

To initiate GDM, females were subjected to a 60% calories-by-fat diet (XIETONG) from week 4 to week 10 of age, after which the high fat diet was continued through pregnancy. Aged 10 weeks, mice were mated overnight. Gestational day 0.5 (GD 0.5) was marked by the presence of a vaginal plug. Apart from weight measurements and cage changes at GD 9.5, pregnant females were not disturbed. GDM mice were assigned into five groups (exosomes, 10 mg/kg): normal mice with 0.2 mL of PBS, GDM mice with PBS, GDM mice with C-mUMSC-EXOs, GDM mice with G-mUMSC-EXOs, and GDM mice with si-circ-G-UMSC-EXOs, injected via tail vein at GD 7 and 13 days. On GD 18, mice body weights were determined by an electronic scale. Blood samples were obtained via tail veins to assess blood glucose levels using a blood glucose meter (Accu-Check active tset strips, Roche). Then, mice were fasted for a specified time for the insulin- and glucose-tolerance tests.

### 2.12. Oral Glucose Tolerance Test (OGTT) and Insulin Tolerance Tests (IPITTs)

For OGTTs, on GD 18, after fasting for 6 h, rats were fasted overnight and intragastrically administered with glucose (2 g/kg bw). Levels of blood glucose were evaluated at 0, 30, 60, 90, and 120 min post-administration. IPITTs were conducted by the intraperitoneal injection of glucose (2 g/kg bw) into rats immediately followed by insulin (2 IU/kg bw) administration. Levels of blood glucose were evaluated at 0, 30, 60, 90, and 120 min post-administration.

### 2.13. Glucose and Glycogen Content Assay

Glucose levels of extracted cells or tissues were evaluated by a high sensitivity glucose assay kit (Sigma-Aldrich) as recommended. Briefly, in the presence of glucose, the formed fluorometric product is proportional to glucose levels. For every sample, 1 and 10 *μ*L were reacted in duplicates with the final volume of the reaction mixture being 100 *μ*L in the 96-well plates. The microplate reader (Thermo fisher, excitation: 535 nm, emission: 587 nm) was used to measure fluorescence. Glycogen content detection was performed using glycogen assay kit (Solarbio) in accordance with instruction from the manufacturer. Briefly, 0.75 mL extract buffer was added into10 mL tube with 0.2 g cell sample, incubated with boiling water bath for 20 min, and then centrifuged at 8000 *g* for 10 min. OD value of 200 *μ*L of supernatant was tested with a microplate reader at 620 nm.

### 2.14. Hematoxylin-Eosin (H&E) Staining

Fresh mice liver tissues were fixed in paraformaldehyde (4%), dehydrated gradually, paraffin-embedded, sliced into 4 *μ*m-thick sections, and H&E (Baso) stained for cellular structure visualization by microscopy.

### 2.15. RNA Extraction and Quantitative Real-Time Polymerase Chain Reaction (RT-qPCR)

The extraction of total RNAs from cells, exosomes, or frozen tissues was done using the Trizol reagent (Takara) and quantified. CircRNAs, miRNAs, and mRNAs were reverse transcribed using the PrimeScript™ RT reagent Kit with gDNA Eraser (TakaRa) as instructed by the manufacturer. Relative quantities of mRNA and circRNAs were determined by the 2^−ΔΔCT^ method with glyceraldehyde-3-phosphate dehydrogenase (GAPDH) as the internal control. However, miRNAs were normalized to the levels of the internal control U6 by the 2^−ΔΔCT^. RT-qPCR was conducted using the ABI step one system with SYBR green (TakaRa) to determine the levels of targets. The detecting primers for circRNAs were designed based on its head to tail junction. Supplementary Table S1 shows the primer sequences.

### 2.16. Western Blot

Whole-cell lysates were prepared from L-02 cells and liver tissue after treatment to collect protein. The proteins were then separated by sodium dodecyl sulfate polyacrylamide gel electrophoresis gel (SDS-PAGE), transferred to polyvinylidene fluoride (PVDF, Millipore, USA) membranes using an electroporation system, and blocked with 5% skim milk, after which membranes were incubated overnight at 4°C in the presence of appropriate specific primary antibodies: anti-CD81 (Abcam, ab79559), anti-CD63 (Abcam, ab217345), anti-IRS-1 (CST, 3407S), anti-p-IRS-1 (CST, 2385S), anti-G6PC2 (BIOSS, bs-22837R), and anti-GAPDH (Zen-bio, 200306-7E4), followed by incubation in the presence of a horseradish peroxidase-conjugated secondary antibody. GAPDH was used as the internal reference for normalization. Chemiluminescence (Bio-rad) was used to detect the bands, while the ImageJ software (Rockville) was used for analysis.

### 2.17. Statistical Analysis

Data are shown as the means ± SD. SPSS ver. 22 software (IBM) was used for statistical analyses, while the GraphPad Prism 7 software (La Jolla) was used to acquire images. Comparisons of means between samples was done by a two-t4646d student's *t*-test, while differences between multiple groups were compared by one-way ANOVA. *P* < 0.05 was the significance threshold (^*∗*^*P* < 0.05, ^*∗∗*^*P* < 0.01, ^*∗∗∗*^*P* < 0.001, and ^*∗∗∗∗*^*P* < 0.0001).

## 3. Results

### 3.1. Identification of hUMSCs and hUMSC-EXOs and Characterization of hsa_circ_0046060

**Figure 1:**
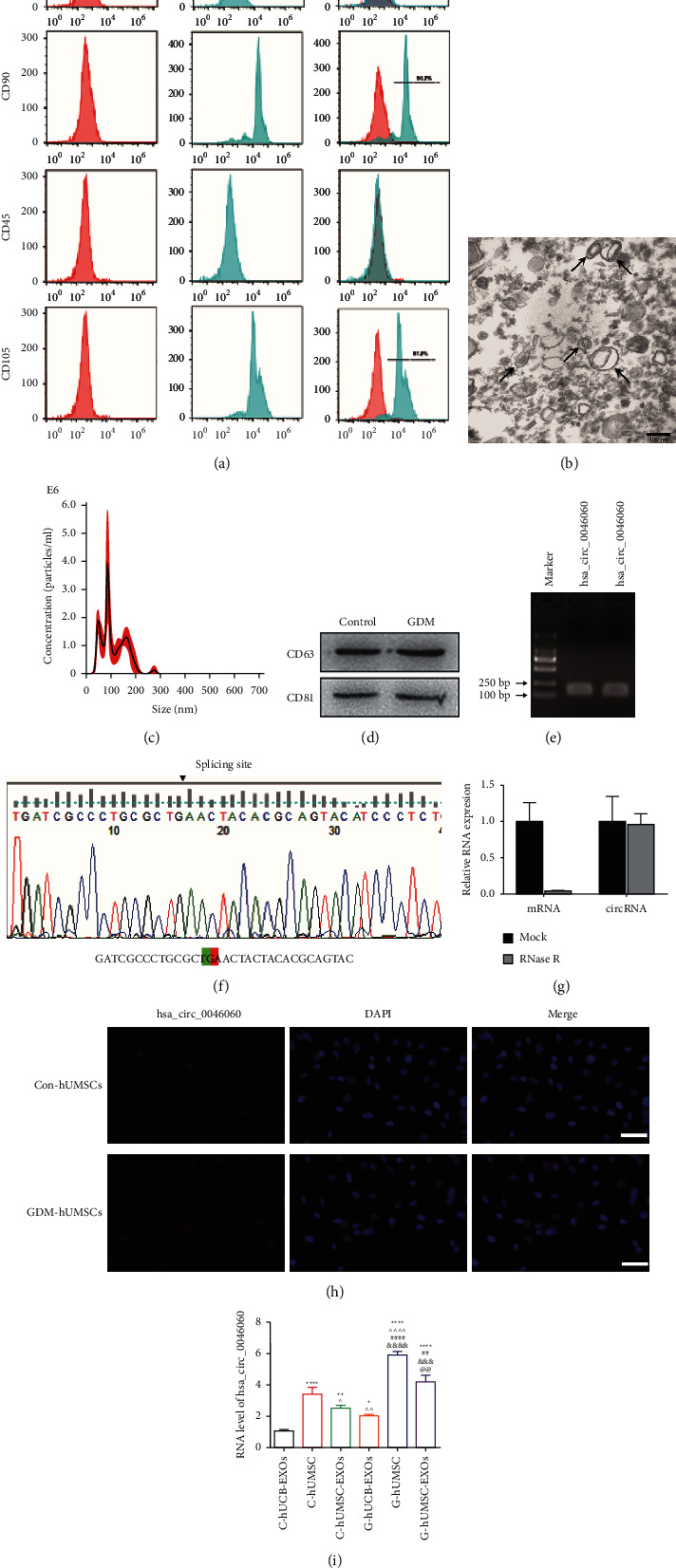
The identification of hUMSCs and hUMSC-EXOs and characterization of hsa_circ_0046060. (a) Flow cytometry analysis showing high expression levels of CD90 and CD105 and negative expression of CD45 and CD34 in hUMSCs. (b) Representative TEM micrographs of hUMSC-Exos characterization. Scale bar: 100 nm. (c) NTA indicating the particle size and concentration of exosomes. (d) Western blot analysis indicating expression of specific markers of huMSC-EXOs. (e) Agarose gel electrophoresis photograph of RT-qPCR assay showing amplification of hsa_circ_0046060. (f) Sanger sequencing analysis using specific primers for hsa_circ_0046060 indicating the splicing site. (g) FISH assay showing localization of hsa_circ_0046060 in the cytoplasm of hUMSCs. (h) RT-qPCR analysis of has_ circ_0046060 and its host gene mRNA treated with or without RNase R Scale bar: 50 *μ*m. (i) has_circ_0046060 expression levels in C-hUCB-EXOs, C-hUMSC, C-hUMSC-EXOs, G-hUCB-EXOs, G-hUMSC and G-hUMSC-EXOs as determined by qRT-PCR (^*∗*^*P* < 0.05, ^*∗∗*^*P* < 0.01, ^*∗∗∗∗*^*P* < 0.0001, vs. C-hUCB-EXOs; ^ *P* < 0.05, ^  ^ *P* < 0.01, ^  ^  ^  ^ *P* < 0.0001, vs. C-hUMSC; ^##^*P* < 0.01, ^####^*P* < 0.0001, vs. C-hUMSC-EXOs; ^&&&^*P* < 0.001, ^&&&&^*P* < 0.001, vs. G-hUCB-EXOs; ^@@^*P* < 0.01, G-hUMSC vs. G-hUMSC-EXOs).

Flow cytometry assay results showed that most hUMSCs highly expressed CD105 (96.2%) and CD90 (97.8%), whereas they exhibited low expression levels of CD45 and CD34, which confirmed hUMSC identity (Figure 1(a)). Vesicle-like features of exosomes were explored using transmission electron microscopy (TEM) to determine the morphology, and the results showed a size of 30–150 nm (Figure 1(b)). NTA results demonstrated that isolated EVs were approximately 100 nm in diameter, and the size of most EVs was below 200 nm, indicating the successful isolation of exosomes (Figure 1(c)). Western blotting analysis showed the expression of exosomal markers, including CD63 and CD81 proteins, in hUMSC-EXOs obtained from control and GDM patients (Figure 1(d)). Therefore, they were referred to as hUMSC-EXOs and used in subsequent experiments.

Agarose gel electrophoresis assay was conducted to evaluate RT-qPCR products of hsa_circ_0046060 with a length of 323 bp (Figure 1(e)), and Sanger sequencing was performed to confirm the splicing site (Figure 1(f)). These findings indicated the circularity of hsa_circ_0046060. Moreover, endogenous hsa_circ_0046060 was found resistant to RNase *R* digestion, however, the expression of the parent gene RPTOR was significantly downregulated (Figure 1(h)). Further analysis through FISH assay of hUMSCs of controls and GDM showed that hsa_circ_0046060 was mainly localized in the cytoplasm (Figure 1(h)). Furthermore, RT-qPCR results showed that hsa_circ_0046060 was expressed in all groups, including control-human umbilical cord exosomes (C-hUCB-EXOs), C-hUMSC, C-hUMSC-EXOs, G-hUCB-EXOs, G-hUMSC, and G-hUMSC-EXOs, and the expression was significantly upregulated in G-hUCB-EXOs compared with C-hUCB-EXOs, which is consistent with the previous microarray results (Figure 1(i)). Notably, hsa_circ_0046060 was significantly upregulated in hMUSCs relative to the expression level in hUCB. The analysis of RT-qPCR data showed that GDM patients exhibited the significant upregulation of hsa_circ_0046060 in hMUSCs-EXOs compared with controls. These findings indicate that hsa_circ_0046060 is a circRNA mainly localized in the cytoplasm and highly expressed in hUMSCs and hMUSCs-EXOs derived from control subjects and GDM patents.

### 3.2. Knock down of GDM-hUMSC Exosomal hsa_circ_0046060 Rescues Intracellular Glucose Uptake *In Vitro*

**Figure 2:**
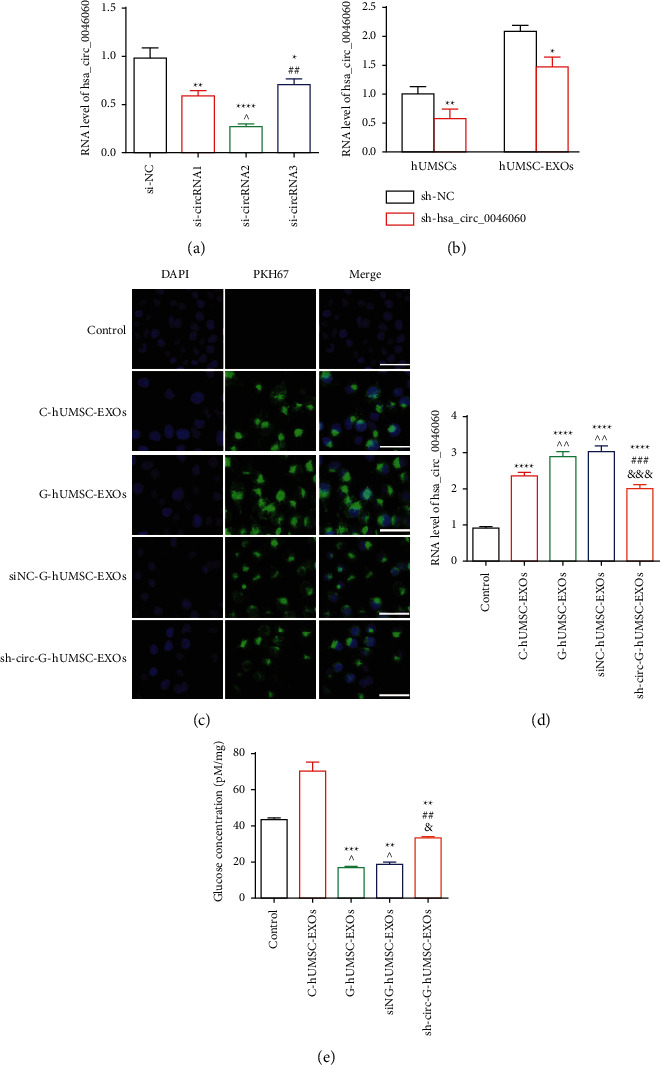
Hsa_circ_0046060 inhibition reversed the effect of GDM-derived hUMSC exosome on intracellular glucose concentration. (a) Inhibitory effects of three candidate siRNAs targeting hsa_circ_0046060 as determined by RT-qPCR ^*∗*^*P* < 0.05, ^*∗∗*^*P* < 0.01, ^*∗∗∗∗*^*P* < 0.0001, vs. siNC; ^^^*P* < 0.05, vs. si-circRNA1; ^##^*P* < 0.01, si-circRNA2 vs. si-circRNA3). (b) Effect of lentiviral system on hsa_circ_0046060 expression level in hUMSCs and hUMSC-EXOs as determined by RT-qPCR. ^*∗*^*P* < 0.05, ^*∗∗*^*P* < 0.01. (c) Fluorescence microscopy showing intake of PKH67 labeled exosomes into L-02 cells after 24 h treatment. Scale bar = 20 *μ*m. (d) Levels of hsa_circ_0046060 were determined using RT-qPCR after treatment with different huMSC-EXOs (^*∗*^*P* < 0.05, ^*∗∗*^*P* < 0.01, ^*∗∗∗∗*^*P* < 0.0001, vs. control; ^  ^ *P* < 0.01, vs. C-hUMSC-EXOs; ^###^*P* < 0.001, vs. G-hUMSC-EXOs; ^&&&^*P* < 0.001, siNC-G-hUMSC-EXOs vs. sh-circ-G-hUMSC-EXOs). (e) Glucose level in L-02 cells as determined using the glucose content assay kit (^*∗∗*^*P* < 0.01, ^*∗∗∗∗*^*P* < 0.0001, vs. control; ^^^*P* < 0.05, vs. C-hUMSC-EXOs; ^##^*P* < 0.01, vs. G-hUMSC-EXOs; ^&^*P* < 0.05, siNC-G-hUMSC-EXOs vs. sh-circ-G-hUMSC-EXOs).

RT-qPCR results showed that the three siRNA, si-circRNA1, si-circRNA2, and si-circRNA3 significantly inhibited the expression of hsa_circ_0046060 compared with the siNC group. Notably, si-circRNA2 exhibited the highest suppression effect relative to the other siRNAs (Figure 2(a)). Therefore, si-circRNA2 was used for the construction of a stable interference system for hsa_circ_0046060 by lentiviral transduction (sh-hsa_circ_0046060) in hUMSCs. RT-qPCR results showed that sh-hsa_circ_0046060 treatment significantly downregulated hsa_circ_0046060 expression in hUMSCs and hUMSC-EXOs (Figure 2(b)). Fluorescence microscopy with PKH67 labeling indicated the intake of hUMSC-EXOs derived from control and GDM donors into the cytoplasm of L-02 cells (Figure 2(c)). As shown in Figure 2(d), expression levels of hsa_circ_0046060 were higher, following hUMSC-EXOs treatment, and sh-circ-G-hUMSC-EXOs significantly downregulated its expression. Glucose cellular uptake assay was performed to further explore the effect of exosomal hsa_circ_0046060 on the uptake of L-02 cells. The incubation of C-hUMSC-EXOs significantly increased glucose concentration (Figure 2(e)). However, hUMSC-EXOs from GDM presenting upregulated expression of hsa_circ_0046060 exhibited reduced glucose uptake, which was alleviated by administration of sh-circ-G-hUMSC-EXOs into L-02 cells. In summary, these results showed that increased expression of hsa_circ_0046060 inhibits glucose uptake and knockdown of hsa_circ_0046060 restores glucose uptake in L-02 cells.

### 3.3. Characterization and Expression Profile of mmu_circ_0002819 in GDM Mice

**Figure 3:**
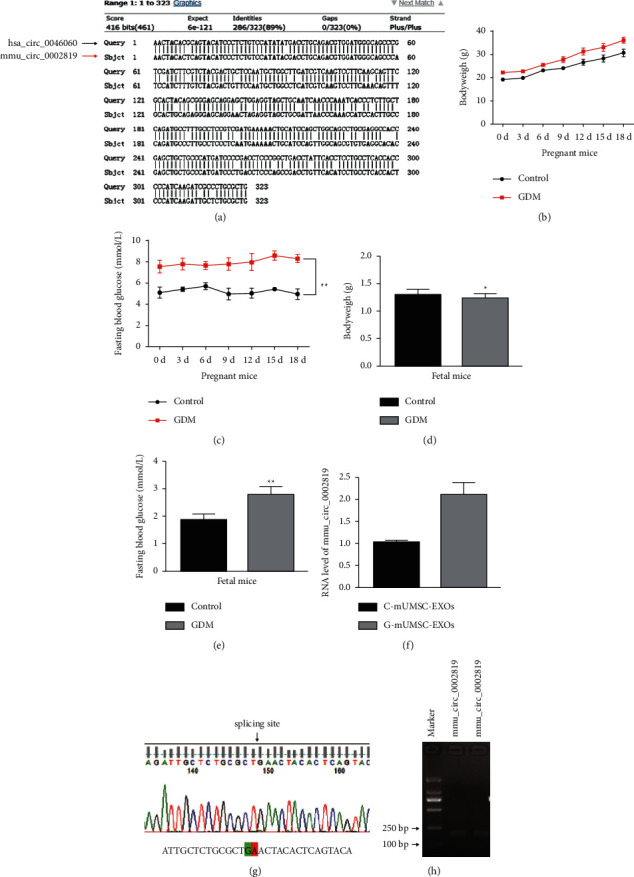
Characterization of hsa_circ_0046060 in hUMSCs and hUMSC-EXOs. (a) Blast analysis to explore homology between human circRNA sequence XXX and mice sequence. (b, d) Bodyweight of pregnant and fetal mice in control and GDM groups as determined by an electronic scale. (c, e) Fasting blood glucose level of pregnant and fetal mice in control and GDM groups as determined using blood glucose test strips. (f) Expression levels of exosomal mmu_circ_0002819 in control and GDM mice as determined using RT-qPCR. (g) Inhibitory effects of three siRNA-mediated knockdown of mmu_circ_0002819 as determined by RT-qPCR assay. (h) Effect of lentiviral sh-mmu_circ_0002819 on mmu_circ_0002819 expression in hUMSCs and hUMSC-EXOs as evaluated by RT-qPCR. (i) Sanger sequence analysis showing the splicing site. (j) RT-qPCR products were evaluated using agarose gel electrophoresis. ^*∗*^*P* < 0.05, ^*∗∗*^*P* < 0.01.

mmu_circ_0002819 is a circRNA generated from the same parent gene as RPTOR in mice. Thus, it has a length of 323 bp. BLAST analysis showed that the sequences of the two circRNAs from human and mice exhibited similarity in 286 bp, accounting for 89% homology (Figure 3(a)), suggesting high sequence conservation between human and mice. Basic physiological characteristics, including body weight and fasting blood glucose of pregnant mice and fetal mice in control and GDM mice, were determined. Body weight of pregnant mice in the GDM group was not significantly different compared with the weight of mice in the control group (Figures 3(b) and 3(c)). Fasting blood glucose level was higher in GDM pregnant mice relative to the level in the control group, indicating the successful establishment of the GDM model. The body weight of fetal mice on day 18 was slightly lower in the GDM group, whereas fasting blood glucose level was higher in GDM mice compared with that of mice in the control group (Figures 3(d) and 3(e)). RT-qPCR results revealed the upregulation of mmu_circ_0002819 expression in GDM mice-derived exosomes compared with the expression in the control group (Figure 3(f)). Sanger sequencing and agarose gel electrophoresis assay were conducted for the characterization of mmu_circ_0002819 in mice (Figures 3(i) and 3(j)). In summary, hsa_circ_0046060 is highly homologous to circRNA mmu_circ_0002819, which is expressed in mUMSC and mUMSC-EXOs derived from both normal and GDM mice.

### 3.4. Silencing of mmu_circ_0002819 Abrogates the Induction of Glucose and Insulin Tolerance in GDM Mouse Model Mediated by Exosomes Derived from GDM-mUMSC

**Figure 4:**
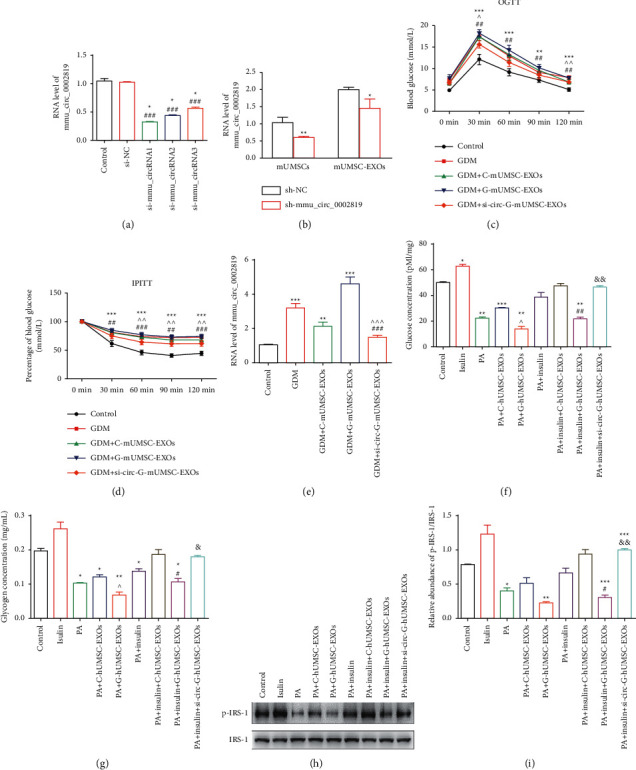
Si-circ-G-mUMSC-EXOs ameliorated glucose and insulin resistance in GDM mice. (a) Inhibitory effects of three potential siRNAs targeting mmu_circ_0002819 as determined by RT-qPCR assay (^*∗*^*P* < 0.05, si-mmu_circRNA1, mmu_si-circRNA2 and mmu_si-circRNA3 vs. Control; ^###^*P* < 0.001, si-mmu_circRNA1, mmu_si-circRNA2 and mmu_si-circRNA3 vs. siNC). (b) Inhibitory effect of sh-mmu_circ_0002819 on mmu_circ_0002819 expression in mUMSCs and mUMSC-EXOs as evaluated by RT-qPCR. ^*∗*^*P* < 0.05, ^*∗∗*^*P* < 0.01 (c) Serum glucose level in mice from the five groups following glucose tolerance test (^*∗∗*^*P* < 0.01, ^*∗∗∗*^*P* < 0.001, vs. Control; ^ *P* < 0.05, ^  ^*P* < 0.01, GDM vs. GDM + si-circ-G-mUMSC-EXOs; ^##^*P* < 0.01, GDM + G-mUMSC-EXOs vs. GDM + si-circ-G-mUMSC-EXOs). (d) Serum glucose level of mice in the five groups following insulin tolerance test (^*∗∗*^*P* < 0.01, ^*∗∗∗*^*P* < 0.001, vs. Control; ^^^*P* < 0.05, ^^^^*P* < 0.01, GDM vs. GDM + si-circ-G-mUMSC-EXOs; ^##^*P* < 0.01, ^###^*P* < 0.001, GDM + G-mUMSC-EXOs vs. GDM + si-circ-G-mUMSC-EXOs). (e) Expression level of mmu_circ_0002819 in mice liver as evaluated using RT-qPCR (^*∗∗*^*P* < 0.01, ^*∗∗∗*^*P* < 0.001, vs. Control; ^  ^  ^*P* < 0.001, GDM vs. GDM + si-circ-G-mUMSC-EXOs; ^###^*P* < 0.001, GDM + G-mUMSC-EXOs vs. GDM + si-circ-G-mUMSC-EXOs). (f) Glucose concentration as determined using Glucose Assay kit (^*∗*^*P* < 0.05, ^*∗∗*^*P* < 0.01, ^*∗∗∗*^*P* < 0.001, vs. Control; ^^^*P* < 0.05, PA + C-hUMSC-EXOs vs. PA + G-hUMSC-EXOs; ^##^*P* < 0.01, PA + insulin + C-hUMSC-EXOs vs. PA + insulin + G-hUMSC-EXOs; ^&&^*P* < 0.01, PA + insulin + G-hUMSC-EXOs vs. PA + insulin + si-circ-G-hUMSC-EXOs). (g) Glycogen levels as determined using Glycogen Content Assay Kit (^*∗*^*P* < 0.05, ^*∗∗*^*P* < 0.01, vs. Control; ^^^*P* < 0.05, PA + C-hUMSC-EXOs vs. PA + G-hUMSC-EXOs; ^#^*P* < 0.05, PA + insulin + C-hUMSC-EXOs vs. PA + insulin + G-hUMSC-EXOs; ^&^*P* < 0.05, PA + insulin + G-hUMSC-EXOs vs. PA + insulin + si-circ-G-hUMSC-EXOs). (h) Expression levels of p-IRS-1 and IRS-1 in the L-02 cells as determined by western blot. (i) Quantification of p-IRS-1 and IRS-1 in different groups (^*∗*^*P* < 0.05, ^*∗∗*^*P* < 0.01, vs. Control; ^#^*P* < 0.05, PA + insulin + C-hUMSC-EXOs vs. PA + insulin + G-hUMSC-EXOs; &&*P* < 0.01, PA + insulin + G-hUMSC-EXOs vs. PA + insulin + si-circ-G-hUMSC-EXOs).

Three alternative siRNAs were designed, and the one exhibiting most suppression effect on mmu_circ_0002819 expression in mUMSCs was selected for subsequent analysis. The three siRNAs (si-mmu_circRNA1, si-mmu_circRNA2, and si-mmu_circRNA3) downregulated the expression of mmu_circ_0002819. Notably, si-mmu_circRNA1 showed the most significant suppression effect. Thus, it was selected for subsequent studies (Figure 4(a)). Lentiviral-wrapped si-mmu_circRNA1 (sh-mmu_circ_0002819) was constructed to evaluate the interference effect in mUMSCs and mUMSC-EXOs. RT-qPCR results showed that sh-mmu_circ_0002819 transduction significantly downregulated the expression of mmu_circ_0002819 in mUMSCs and mUMSC-EXOs (Figure 4(b)). C-mUMSC-EXOs, G-mUMSC-EXOs, and si-circ-G-mUMSC-EXOs did not significantly affect the body weight and fasting blood glucose level of pregnant mice compared with the control (Supplementary Figures S1(a) and S1(b)). Notably, increased fasting blood glucose level of fetal mice was observed in the GDM mouse model after administration with PBS, C-mUMSC-EXOs, G-mUMSC-EXOs, and si-circ-G-mUMSC-EXOs compared with the level in control mice (Supplementary Figure S1(c)). However, the body weight was not affected (Supplementary Figure S1(d)). The body weight of fetal mice treated with G-mUMSC-EXOs was significantly lower compared with that of other groups. OGTT results showed that the administration of C-mUMSC-EXOs and si-circ-G-mUMSC-EXOs improved glucose metabolism in GDM mice (Figure 4(c)). Furthermore, IPITTs results indicated that C-mUMSC-EXOs and si-circ-G-mUMSC-EXOs administration significantly ameliorated insulin sensitivity compared with the control (Figure 4(d)). It implies that the inhibition of mmu_circ_0002819 improves dysregulated glucose and insulin function.

Furthermore, the expression profile of mmu_circ_0002819 was explored in mice liver to determine whether mmu_circ_0002819-mediated regulation of glucose metabolism is involved in the biological processes of the liver. RT-qPCR results showed that the expression of mmu_circ_0002819 was upregulated in GDM and GDM + G-mUMSC-EXOs groups, whereas G-mUMSC-EXOs and si-circ-G-mUMSC-EXOs groups exhibited downregulated mmu_circ_0002819 expression (Figure 4(e)). Subsequently, glucose content analysis and glycogen synthesis assay were performed using human L-02 cells, showing that insulin improved intracellular glucose concentration, however, intracellular glucose concentration was reduced in palmitic acid (PA)-induced insulin-resistant L-02 cells (Figure 4(f)). Notably, C-hUCMSC-EXOs and si-circ-G-hUMSC-EXOs ameliorated PA-induced and G-hUCMSC-EXOs-induced inhibition of intracellular glucose level. The analysis of glycogen levels showed that insulin induced glycogen synthesis, which was impaired by PA. Furthermore, C-hUCMSC-EXOs and si-circ-G-hUMSC-EXOs promoted glycogenesis in PA-induced insulin-resistant L-02 cells (Figure 4(g)). This finding implies that accumulated intracellular glucose was mainly metabolized through the glycogenesis pathway, and this process was regulated by hsa_circ_0046060. Furthermore, the effect of exosomal hsa_circ_0046060 on the insulin receptor substrate-1 (IRS-1) pathway was evaluated. Western blot results showed that C-hUCMSC- EXOs and si-circ-G-hUMSC-EXOs modulated the expression of p-IRS-1 to IRS-1. The incubation of cells with insulin promoted the phosphorylation of IRS-1. The addition of PA suppressed the phosphorylation of IRS-1, and G-hUCMSC- EXOs showed significantly higher inhibitory effect. However, the reduced activation of IRS-1 was rescued by the administration of C-hUCMSC- EXOs and si-circ-G-hUMSC-EXOs (Figures 4(h) and 4(i)). These results indicated that the knockdown of hsa_circ_0046060 improved insulin sensitivity in GDM mice and insulin-resistant cells through the insulin receptor-mediated pathway.

### 3.5. Hsa_circ_0046060 Upregulates G6PC2 Expression by Sponging Hsa-miR-338-3p

**Figure 5:**
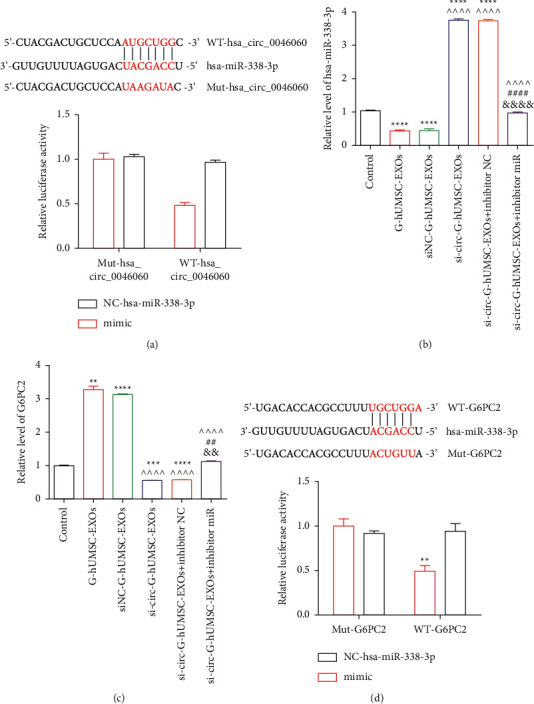
Hsa-miR-338-3p was directly bound to hsa_circ_0046060 and it regulated G6PC2 expression. (a) hsa-miR-338-3p targets the 3′-UTR sequence of hsa_circ_0046060. Luciferase reporter assay was performed to verify the interaction between hsa_circ_0046060 and hsa-miR-338-3p in L-02 cells. ^*∗∗*^*P* < 0.01. Effects of hsa-miR-338-3p inhibitor on the expression of (b) hsa-miR-338-3p (^*∗∗∗∗*^*P* < 0.0001, vs. Control; ^  ^  ^  ^ *P* < 0.0001, vs. siNC-G-hUMSC-EXOs; ^####^*P* < 0.0001, si-circ-G-hUMSC-EXOs vs. si-circ-G-hUMSC-EXOs + inhibitor miR; ^&&&&^*P* < 0.0001, si-circ-G-hUMSC-EXOs vs. si-circ-G-hUMSC-EXOs + inhibitor miR) and (c) G6PC2 (^*∗∗*^*P* < 0.01, ^*∗∗∗*^*P* < 0.001, ^*∗∗∗∗*^*P* < 0.0001, vs. Control; ^  ^  ^  ^*P* < 0.0001, vs. siNC-G-hUMSC-EXOs; ^##^*P* < 0.01, si-circ + G-hUMSC-EXOs vs. si-circ-G-hUMSC-EXOs + inhibitor miR; ^&&^*P* < 0.01, si-circ-G-hUMSC-EXOs vs. si-circ-G-hUMSC-EXOs + inhibitor miR) as determined by RT-qPCR assay. (d) hsa-miR-338-3p targets the 3′-UTR sequence of G6PC2. The relative luciferase activity was evaluate to verify the interaction between G6PC2 and hsa-miR-338-3p in L-02 cells. ^*∗∗*^*P* < 0.01.

CircRNAs located in the cytoplasm mainly act as miRNA sponges to regulate gene expression. Bioinformatic analysis using CircInteractome tool (https://circinteractome.nia.nih.gov/) revealed that hsa_circ_0046060 potentially binds to four candidate miRNAs, including hsa-miR-338-3p, hsa-miR-142-3p, hsa-miR-369-5p, and hsa-miR-370-3p. RT-qPCR results showed a significant downregulation of hsa-miR-338-3p (Supplementary Figure S2(a)), hsa-miR-142-3p (Supplementary Figure S2(b)), hsa-miR-369-5p (Supplementary Figure S2(c)), and hsa-miR-370-3p (Supplementary Figure S2(d)) after the incubation of cells with G-hUCMSC-EXOs and siNC-G-hUMSC-EXOs in L-02 cells. On the contrary, si-circ-G-hUMSC-EXOs upregulated the expression of these miRNAs, with hsa-miR-338-3p that was selected for subsequent functional studies, exhibiting the most significant variation. The findings using luciferase reporter assay indicated that hsa-miR-338-3p mimics significantly downregulated relative Renilla luciferase gene activity of WT-hsa_circ_0046060 rather than that of NC-hsa-miR-338-3p (Figure 5(a)), implying that hsa-miR-338-3 interacted with hsa_circ_0046060. Furthermore, TargetScan (https://www.targetscan.org/vert_72/) tool was used to predict the downstream targets of hsa-miR-338-3p, and the result showed that PTEN and G6PC2 may interact with hsa-miR-338-3p. Subsequently, RT-qPCR results showed that hsa-miR-338-3p inhibitor effectively downregulated the expression of hsa-miR-338-3p in L-02 cells (Figure 5(b)). Notably, PTEN (Supplementary Fig.  S2(e)) and G6PC2 (Figure 5(c)) exhibited completely opposite expression patterns relative to hsa-miR-338-3p level following the same treatment. G-hUCMSC-EXOs and siNC- G-hUCMSC-EXOs significantly upregulated the expression of PTEN and G6PC2 compared with control group, whereas the administration of si-circ-G-hUMSC-EXOs alone and coincubation with inhibitor NC reversed the elevated expression. Hsa-miR-338-3p inhibitor abrogated the effect of the knockdown of hsa_circ_0046060. Luciferase reporter assays showed that Renilla luciferase activity was significantly lower in the WT-G6PC2 + hsa-miR-338-3p mimics group compared with that in the WT-G6PC2 + mimics NC group (Figure 5(d)). These findings indicate that hsa_circ_0046060 negatively targets hsa-miR-338-3p to upregulate G6PC2 expression, thus modulating glucose metabolism and insulin sensitivity.

### 3.6. Exosomal hsa_circ_0046060 Derived from hUMSC Targets G6PC2 to Modulate Glucose Homeostasis and Induce Insulin Resistance through Hsa-miR-338-3p

**Figure 6:**
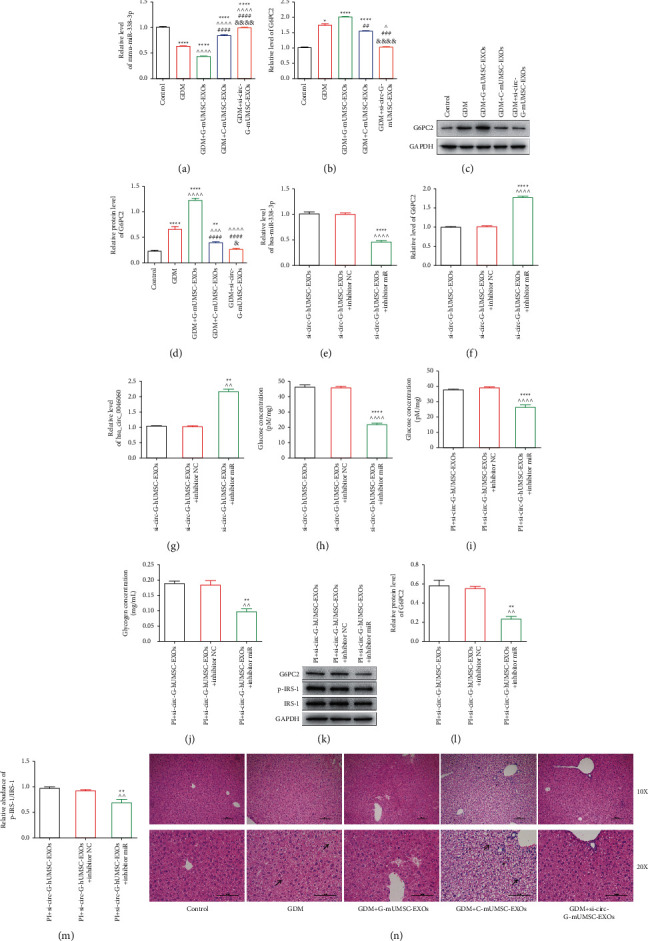
Exosomal hsa_circ_0046060 of hUMSC regulates glucose homeostasis and induces insulin resistance through hsa-miR-338-3p in vitro and in vivo. (a) mmu-miR-338-3p expression level (^*∗∗∗∗*^*P* < 0.0001, vs. Control; ^  ^  ^  ^ *P* < 0.0001, vs. GDM; ^####^*P* < 0.0001, vs. GDM + G-mUMSC-EXOs; ^&&&&^*P* < 0.0001, GDM + C-mUMSC-EXOs vs. GDM + si-circ-G-mUMSC-EXOs) and (b) G6PC2 mRNA expression level (^*∗*^*P* < 0.05, ^*∗∗∗∗*^*P* < 0.0001, vs. Control; ^ *P* < 0.05, GDM vs. GDM + si-circ-G-hUMSC-EXOs; ^##^*P* < 0.01, ^###^*P* < 0.001, vs. GDM + G-mUMSC-EXOs; ^&&&&^*P* < 0.0001, GDM + C-mUMSC-EXOs vs. GDM + si-circ-G-hUMSC-EXOs) in GDM mice administered with PBS, C-mUMSC-EXOs, G-mUMSC-EXOs and si-circ-G-mUMSC-EXOs as evaluated by RT-qPCR. U6 was used as an internal control. (c, d) Protein expression levels of G6PC2 in GDM mice administrated with PBS, C-mUMSC-EXOs, G-mUMSC-EXOs and si-circ-G-mUMSC-EXOs as determined by western blot. GAPDH was used as an internal control (^*∗∗*^*P* < 0.01, ^*∗∗∗*^*P* < 0.001, ^*∗∗∗∗*^*P* < 0.0001, vs. Control; ^  ^  ^ *P* < 0.001, ^  ^  ^  ^ *P* < 0.0001, vs. GDM; ^####^*P* < 0.0001, vs. GDM + G-mUMSC-EXOs; ^&^*P* < 0.05, GDM + C-mUMSC-EXOs vs. GDM + si-circ-G-mUMSC-EXOs). Expression levels of (e) hsa-miR-338-3p (^*∗∗∗∗*^*P* < 0.0001, vs. si-circ-G-hUMSC-EXOs; ^  ^  ^  ^ *P* < 0.0001, vs. si-circ-G-hUMSC-EXOs + inhibitor NC), (f) G6PC2 (^*∗∗∗∗*^*P* < 0.0001, vs. si-circ-G-hUMSC-EXOs; ^  ^  ^  ^ *P* < 0.0001, vs. si-circ-G-hUMSC-EXOs + inhibitor NC) and (g) hsa_circ_0046060 (^*∗∗*^*P* < 0.01, vs. si-circ-G-hUMSC-EXOs; ^  ^ *P* < 0.01, vs. si-circ-G-hUMSC-EXOs + inhibitor NC) following treatment with inhibitor NC and hsa-miR-338-3p inhibitor of si-circ-G-hUMSC-EXOs in L-02 cells as determined by RT-qPCR assay. Glucose concentration: (h) in si-circ-G-hUMSC-EXOs group alone and with inhibitor NC and hsa-miR-338-3p inhibitor (^*∗∗∗∗*^*P* < 0.0001, vs. si-circ-G-hUMSC-EXOs; ^^^^^^*P* < 0.0001, vs. si-circ-G-hUMSC-EXOs + inhibitor NC), and (i) PA-induced insulin resistance cell model alone and with inhibitor NC and hsa-miR-338-3p inhibitor of L-02 cells (PI : PA + insulin, ^*∗∗∗*^*P* < 0.001, vs. PI + si-circ-G-hUMSC-EXOs; ^  ^  ^*P* < 0.001, vs. PI + si-circ-G-hUMSC-EXOs + inhibitor NC) as determined using glucose content assay kit. (j) Glycogen levels in insulin resistance model of L-02 cells as determined using Glycogen Content Assay Kit (PI : PA + insulin, ^*∗∗*^*P* < 0.01, vs. PI + si-circ-G-hUMSC-EXOs; ^  ^ *P* < 0.01, vs. PI + si-circ-G-hUMSC-EXOs + inhibitor NC). (k) Protein expression levels of (l) G6PC2 (PI : PA + insulin, ^*∗∗*^*P* < 0.01, vs. PI + si-circ-G-hUMSC-EXOs; ^  ^ *P* < 0.01, vs. PI + si-circ-G-hUMSC-EXOs + inhibitor NC), (m) p-IRS-1 and IRS-1 (PI : PA + insulin, ^*∗∗*^*P* < 0.01, vs. PI + si-circ-G-hUMSC-EXOs; ^  ^*P* < 0.01, vs. PI + si-circ-G-hUMSC-EXOs + inhibitor NC) in PA-induced insulin resistance cell model alone and with inhibitor NC and hsa-miR-338-3p inhibitor of in L-02 cells as determined by western blot analysis. (n) Morphological changes of liver from GDM mice as evaluated by HE staining. Scale bar = 100 *μ*m.

The expression of mmu-miR-338-3p, a homologue of hsa-miR-338-3p, and G6PC2 in mice liver was explored after administration with PBS, C-mUMSC-EXOs, G-mUMSC-EXOs, and si-circ-G-mUMSC-EXOs to evaluate the regulatory roles of hsa_circ_0046060/hsa-miR-338-3p/G6PC2 axis in GDM mice. The expression of mmu-miR-338-3p in the liver was downregulated in GDM and GDM + G-mUMSC-EXOs groups, whereas C-mUMSC-EXOs and si-circ-G-mUMSC-EXOs groups exhibited unregulated mmu-miR-338-3p expression (Figure 6(a)), which was opposite but functionally explains the results on the expression of mmu_circ_0002819. RT-qPCR results showed a similar G6PC2 expression profile to that of mmu_circ_0002819 in the five different groups, indicating the potential regulatory mechanism underlying hsa_circ_0046060/hsa-miR-338-3p/G6PC2 pathway (Figure 6(b)). Moreover, western blot results showed that G-mUMSC-EXOs upregulated the expression of G6PC2 *in vivo*, and the silencing of mmu_circ_0002819 reversed this phenotype (Figures 6(c) and 6(d)). RT-qPCR results indicated that all three siRNAs (si-G6PC2-1, si-G6PC2-2, and si-G6PC2-3) effectively downregulated the expression of G6PC2 in L-02 cells (Supplementary Figure S3(a)), whereby si-G6PC2-1 exhibited the most significant inhibition. Western blot results showed that si-G6PC2-1 significantly inhibited G6PC2 protein expression in L-02 cells (Supplementary Figures S3(b) and S3(c)).

Intracellular glucose content assays were conducted using L-02 cells with or without si-G6PC2-1 to explore whether G6PC2 was involved in the regulation of glucose metabolism. Western blot results showed that si-G6PC2-1 significantly increased glucose concentration compared with the siNC group (Supplementary Figure S3(d)). Rescue experiments were performed using L-02 cells assigned to three groups, including si-circ-G-hUMSC-EXOs, si-circ-G-hUMSC-EXOs + inhibitor NC, and si-circ-G-hUMSC-EXOs + hsa-miR-338-3p inhibitor to further explore the interaction among hsa_circ_0046060, hsa-miR-338-3p and G6PC2. RT-qPCR results revealed that hsa-miR-338-3p expression was significantly downregulated in si-circ-G-hUMSC-EXOs + hsa-miR-338-3p inhibitor group, whereas there was no effect on the other two groups (Figure 6(e)). The expressions of hsa_circ_0046060 (Figure 6(f)) and G6PC2 (Figure 6(g)) were upregulated in si-circ-G-hUMSC-EXOs + hsa-miR-338-3p inhibitor group. Further analysis showed that hsa-miR-338-3p inhibitors significantly decreased glucose concentration in L-02 cells combined with si-circ-G-hUMSC-EXOs compared with the control group and si-circ-G-hUMSC-EXOs + inhibitor NC group (Figure 6(h)). Furthermore, the knockdown of hsa-miR-338-3p significantly increased glucose concentration in the insulin resistance model (Figure 6(i)). Notably, miR-338-3p-inhibitor exerted significant suppression on glycogen synthesis compared with the use of si-circ-G-hUMSC-EXOs alone and with inhibitor NC (Figure 6(j)). The findings using western blot assay showed that the incubation of cells with miR-338-3p-inhibitor significantly decreased the activation of IRS-1 (Figure 6(l)) and downregulated the expression of G6PC2 compared with the control and inhibitor-NC treatment groups (Figure 6(m)). These results demonstrated that the amelioration of glucose metabolism and insulin sensitivity through the knockdown of hsa_circ_0046060 can be reversed by the co-action of hsa-miR-338-3p inhibitors through the regulation of G6PC2 expression.

The activation of IRS-1 and morphological changes of liver in GDM mice were evaluated to explore whether exosomal mmu_circ_0002819 affects glucose metabolism process *in vivo*. Western blot results showed that GDM alone or combined with G-mUMSC-EXOs suppressed the activation of IRS-1, and the effect was rescued by the administration of C-mUMSC-EXOs and si-circ-G-mUMSC-EXOs (Supplementary Figure S3(e), S3(f)). HE staining of mice liver exhibited disordered lipid metabolism characterized by microvascular steatosis, hepatocytes partially filled with lipid vacuoles, and abnormal nucleus suspended at the center or squeezed to one side of the cell in GDM and GDM + G-mUMSC-EXOs groups (Figure 6(n)). Notably, si-circ-G-mUMSC-EXOs significantly ameliorated these diabetic liver pathological changes. In summary, the findings indicate that the silencing of exosomal mmu_circ_0002819 *in vivo* restores glucose homeostasis and improves liver function.

## 4. Discussion

Previous studies explored the roles of exosomes secreted by several body fluids, such as hUMSCs. Exosomes are involved in several biological processes, including immune inflammation, tissue repair, neuroprotection, and insulin resistance [23, 25–27]. Although previous studies report that hUMSC-derived exosomes (hUMSC-EXOs) have a therapeutic effect on type 2 diabetes mellitus (T2DM), the component of hUMSC-EXOs that exerts the precise regulatory roles has not been fully elucidated. Exosomes carry various molecular functional molecules, including protein, mRNA, miRNA, and circRNA. We previously identified a number of significantly dysregulated circRNAs in transcriptome profiles, of which hsa_circ_0046060 was identified as a potential candidate for diagnosing and treating GDM. The aim of the present study was to explore the role of hsa_circ_0046060 derived from different sources of hUMSC-EXOs in the regulation of GDM pathogenesis. HUMSCs and hUMSC-EXOs were thus isolated and identified. Moreover, functional experiments showed that GDM-derived UMSC exosomes (G-UMSC-EXOs) aggravated the aberrant glucose metabolism and insulin resistance in human and mice comparing with control-derived UMSC exosomes (C-UMSC-EXOs). In contrast, knocking down exosomal hsa_circ_0046060 and mmu_circ_0002819 ameliorated these effects. Mechanistic studies showed that hsa_circ_0046060 mediated its regulatory role by sponging hsa-miR-338-3p, thus regulating G6PC2 and IRS-1 that are implicated in insulin function and glucose metabolism [28–31]. In summary, the upregulation of hsa_circ_0046060 and mmu_circ_0002819 expression in G-UMSC-EXOs dysregulated glucose uptake and insulin resistance by sponging hsa-miR-338-3p to modulate the activation of IRS-1 and G6PC2 expression, which was reversed through the silencing of hsa_circ_0046060 and/or mmu_circ_0002819, thus restoring normal glucose metabolism and insulin sensitivity.

Accumulating evidence demonstrated that hUMSC-EXOs can ameliorate insulin resistance and alleviate excessive inflammation. Therefore, they play a significant role in treatment for GDM and diabetic complications, including myocardial infarction, diabetic retinopathy, and diabetic nephropathy [32–35]. Based on the results of the present study, hUMSC-EXOs isolated from healthy control and GDM patients, as well as mice, may play a significant role in the development of GDM. The results indicated that the administration of G-UMSC-EXOs dysregulated the hemostasis of glucose and insulin by upregulating the expression of hsa_circ_0046060 and mmu_circ_0002819 compared with C-UMSC-EXOs. Moreover, G6PC2 expression was upregulated, whereas the expression of miR-338-3p was downregulated and the phosphorylation of IRS-1 was suppressed. Most studies that explored the roles of hUMSC-EXOs mainly focused on the advantages of comprehensive alleviation of GDM clinical manifestations. Notably, only a few of the studies have investigated the precise molecular mechanisms underlying the regulation of GDM pathogenesis by hUMSC-EXOs. Sun et al., reported that hUMSC-EXOs exerts therapeutic effects on T2DM by significantly improving hyperglycemia and enhancing insulin sensitivity, mainly through the activation of IRS-1 and AKT, as well as repressing the release of proinflammatory cytokines [27]. HUMSC-EXOs regulates the membrane translocation of glucose transporter-4 (GLUT4) and the activation of enzymes implicated in glucose metabolism to improve glucose uptake and glycogen deposit. In addition, an *in vitro* study on the roles of human umbilical vein endothelial cells-derived exosomal circNRA demonstrated that the expression of circ_0074673 was upregulated in exosomes isolated from GDM patients and in human umbilical vein endothelial cells incubated with exosomes [36]. Notably, the knockdown of circ_0074673 alleviates the suppression of proliferation, migration, and angiogenesis of umbilical vein endothelial cells by directly sponging miR-1200 to target MEOX2. It indicates a potential regulatory and therapeutic role of circ_0074673 for GDM. Furthermore, Chen et al. performed glucose homeostasis and insulin sensitivity experiments and compared hUMSCs with human adipose-derived mesenchymal stem cells (hAMSCs). The findings showed that hAMSCs exhibited more notable adipogenic activities relative to hUMSCs by significantly upregulating glucose uptake and the expression of sirtuin-1 and IRS-1, and downregulating expression of leptin [37]. However, the molecular mechanisms underlying these effects have not been fully elucidated.

Previous studies reported that exosomal circRNAs are involved in the pathological processes of GDM, including glycosylation, trophoblast cell biological dysfunction, lipid metabolism, angiogenesis, and wound healing [14, 38–40]. In current study, hUMSC-derived exosomal hsa_circ_0046060, a novel circRNA that has not been reported before, exhibited significant regulatory effects on glucose metabolism and insulin sensitivity *in vivo* and *in vitro*. Similar to the classic function pattern known as the circRNA-miRNA-mRNA axis, the function of optimal downstream targets, including hsa-miR-338-3p and G6PC2, was explored. Previous findings showed that hsa-miR-338-3p mediates a series of biological process associated with the etiology of GDM, such as gluconeogenesis, hepatic insulin resistance, and vascular endothelial cells apoptosis, by targeting distinct genes, including HIF-1*α*, AATK, PTIP, and PP4R1 [41–44]. The results of the present study indicated that hsa_circ_0046060 downregulated the expression of hsa-miR-338-3p in the GDM model mice. Notably, the silencing of hsa-miR-338-3p abrogated hsa_circ_0046060-mediated aggravation of glucose metabolism and insulin resistance. G6PC2 modulates fasting blood glucose level and susceptibility to type 2 diabetes. Moreover, G6PC2 acts as a negative modulator of basal glucose-induced insulin release [31, 45, 46]. The present findings indicated that G6PC2 exhibited synchronous expression trend to that of hsa_circ_0046060. It follows that hsa_circ_0046060 may regulate glucose transport and insulin sensitivity by upregulating the expression of G6PC2, thereby leading to glucose cycling damage. The suppression of hsa_circ_0046060 can be alleviated by the inhibition of hsa-miR-338-3p, indicating a successive action axis. IRS-1 plays a key role in the regulation of insulin resistance and hepatic glucose metabolism. For instance, the elevated activation of IRS-1 improves insulin sensitivity [47, 48]. The phosphorylation of IRS-1 was determined to explore the role of potential stimulator on insulin function. Consistent with previous studies, the results showed that UMSC-EXOs derived hsa_circ_0046060 repressed IRS-1 activation, resulting in insulin resistance. However, the knockdown of hsa_circ_0046060 in UMSC-EXOs rescued insulin sensitivity. These results indicated that normal UMSC-EXOs markedly improved abnormal conditions during GDM development, whereas GDM-derived UMSC-EXOs play negative regulatory roles on glucose homeostasis and insulin sensitivity. These effects mainly exerted through the upregulation of hsa_circ_0046060-mediated decrease in the activation of IRS-1 by the sponging of hsa-miR-338-3p to modulate G6PC2 expression.

Despite the promising findings, there were several limitations in our study. Comparing to the related studies regarding the regulatory role of UMSCs and circRNAs on GDM pathogenesis, it is found that inflammatory alternation, specific cell types, and cell death pathway that contributed to the development of GDM were explored in other studies. Although functional assays were conducted to determine the pathological manifestations of GDM, including the dysregulated glucose transport and insulin sensitivity, the efforts made to deeply investigate the potential factors or precise molecular pathways that may facilitate the deteriorated and/or ameliorated processes were lacking in present study, e.g., how GLUT4 acts in the regulation of glucose metabolism and whether the severity of GDM is associated with the expression level and function of hsa_circ_0046060. In addition, the present study was subject to a potential methodological bias. Methods used to evaluate glucose uptake were not discriminative enough to determine the exact cause of the dysregulation of glucose absorption. Increase in intracellular glucose concentration may have been because of increased intracellular glucogenesis following treatment with different exosomes. Nevertheless, a further analysis of glycogen deposition revealed that glycogen cycling was the pathway most likely to result in accumulating glucose. It indicated that the strategies used for the analysis of glucose metabolism partly explored glucose uptake into hepatic cells. The comparative analysis of selected circRNAs in human and mice was performed. The empirical results showed that circRNAs in these two species shared high homology and exhibited similar regulatory functions. Further analysis was conducted to explore potential difference in their molecular mechanism, mainly the ambiguous roles of hsa-miR-338-3p in the pathogenesis of GDM. Bioinformatics analysis showed that PTEN is a downstream target of miR-338-3p. The findings showed that PTEN exhibited similar changes to those of G6PC2 following the administration of differentially sourced exosomes. Studies report that PTEN impairs insulin signaling and induces insulin resistance during the pathogenesis of type 2 diabetes [49]. Metabolic organs, including the liver and muscle, exhibit significant increase in insulin sensitivity and glucose metabolism after the knockout of PTEN [50, 51]. However, the mechanism underlying the effect of PTNE on insulin sensitivity should be further explored. PTEN may exert its effects by acting as a downstream target for hsa_circ_0046060 and hsa-miR-338-3p, thus alleviating GDM. However, further experiments should be conducted to confirm this hypothesis.

In conclusion, the present study unveiled that the administration of healthy control-derived exosomes and the silencing of hsa_circ_0046060 can ameliorate the aberrant glucose metabolism and insulin sensitivity by sponging hsa-miR-338-3p to regulate G6PC2 expression and the activation of IRS-1 *in vivo* and *in vitro*. The findings of this study provide a novel strategy and new potential molecular targets for the treatment of GDM.

## Data Availability

The data used to support the findings of this study are available from the corresponding author upon request.

## Supplementary Materials

Supplementary Materials Supplemental table1: showing the primers used by RT-qPCR and small-interfering RNA. Figure S1: basic physiological characteristic of body weight and fasting blood glucose of pregnant mice and fetal mice. The body weight of (a) pregnant and (c) fetal mice was assessed by an electronic weigher in control and GDM mice with PBS, C-mUMSC-EXOs, G-mUMSC-EXOs, and si-circ-G-mUMSC-EXOs and GDM groups (vs. GDM + C-mUMSC-EXOs, ^*∗*^*P* < 0.05, ^*∗∗*^*P* < 0.01, and ^*∗∗∗*^*P* < 0.001). The fasting glucose level of (b) pregnant and (d) fetal mice was measured by blood glucose test strips in control and GDM mice with PBS, C-mUMSC-EXOs, G-mUMSC-EXOs, and si-circ-G-mUMSC-EXOs and GDM groups. ^*∗∗∗*^*P* < 0.001 vs. Control. Figure S2: the relative expression of four candidate miRNAs following different treatment. RT-qPCR assay was performed to detect the level of (a) hsa-miR-338-3p (^*∗*^*P* < 0.05, ^*∗∗∗*^*P* < 0.001 vs. Control; ^^^^*P* < 0.01 G-hUMSC-EXOs vs. si-circ-G-hUMSC-EXOs; ^##^*P* < 0.01, siNC-G-hUMSC-EXOs vs. si-circ-G-hUMSC-EXOs), (b) hsa-miR-142-3p (^*∗∗∗∗*^*P* < 0.0001 vs. Control; ^^^^^^*P* < 0.0001 G-hUMSC-EXOs vs. si-circ-G-hUMSC-EXOs; ^####^*P* < 0.0001, siNC-G-hUMSC-EXOs vs. si-circ-G-hUMSC-EXOs), (c) hsa-miR-369-5p (^*∗∗∗∗*^*P* < 0.0001, vs. Control; ^^^^^^*P* < 0.0001, G-hUMSC-EXOs vs. si-circ-G-hUMSC-EXOs; ^####^*P* < 0.0001, siNC-G-hUMSC-EXOs vs. si-circ-G-hUMSC-EXOs) and (d) hsa-miR-370-3p (^*∗∗*^, *P* < 0.01, ^*∗∗∗*^*P* < 0.001, vs. Control; ^^^^*P* < 0.01 G-hUMSC-EXOs vs. si-circ-G-hUMSC-EXOs; ^##^*P* < 0.01, siNC-G-hUMSC-EXOs vs. si-circ-G-hUMSC-EXOs) with PBS, G-hUMSC-EXOs, siNC-G-hUMSC-EXOs and si-circ-G-hUMSC-EXOs in L-02 cells. (e) RT-qPCR assay was performed to determine the effects of hsa-miR-338-3p inhibitor on the expression of PTEN (^*∗∗*^*P* < 0.01, ^*∗∗∗∗*^*P* < 0.0001 vs. Control; ^^^^^^*P* < 0.0001, vs. siNC + G-hUMSC-EXOs; ^##^*P* < 0.01, si-circ + G-hUMSC-EXOs vs. si-circ + G-hUMSC-EXOs + inhibitor miR; ^&&^*P* < 0.01, si-circ + G-hUMSC-EXOs vs. si-circ + G-hUMSC-EXOs + inhibitor miR). Figure S3 Exosomal hsa_circ_0046060 of hUMSC regulated glucose homeostasis and induce insulin resistance via hsa-miR-338-3p *in vitro* and *in vivo*. (a) RT-qPCR assay determined the inhibitory effects of three potential siRNAs targeting G6PC2 (^*∗∗∗∗*^*P* < 0.0001, vs. Control; ^^^^^^*P* < 0.0001, vs. siNC; ^####^*P* < 0.0001, vs. si-G6PC2-1). (b, c) The protein expression of G6PC2 in L-02 cells by incubation of si-G6PC2-1 was detected by Western blot, ^*∗∗∗*^*P* < 0.001. (d) The glucose concentration was measured in siNC and si-G6CP2-1 groups, respectively, ^*∗∗∗*^*P* < 0.001. (e, f) Expression of p-IRS-1 and IRS-1 in the L-02 cells was detected by Western blot in control, GDM mice administrated with PBS, C-mUMSC-EXOs, G-mUMSC-EXOs and si-circ-G-mUMSC-EXOs (^*∗∗∗*^*P* < 0.001, ^*∗∗∗*^*P* < 0.0001, vs. Control; ^^^^^^*P* < 0.0001, vs. GDM; ^##^*P* < 0.01, vs. GDM + G-mUMSC-EXOs; ^&&&&^*P* < 0.01, vs. GDM + C-mUMSC-EXOs).
