# Supplementary material for: Exosomal Circular RNA hsa_circ_0046060 of Umbilical Cord Mesenchymal Stromal Cell Ameliorates Glucose Metabolism and Insulin Resistance in Gestational Diabetes Mellitus via the miR-338-3p/G6PC2 Axis
Source: Int J Endocrinol. 2022 Jun 11;2022:9218113. doi: 10.1155/2022/9218113 (PMC9206588; doi:10.1155/2022/9218113)
Supplement: Supplementary Materials — Supplemental table1: showing the primers used by RT-qPCR and small-interfering RNA. Figure S1: basic physiological characteristic of body weight and fasting blood glucose of pregnant mice and fetal mice. The body weight of (a) pregnant and (c) fetal mice was assessed by an electronic weigher in control and GDM mice with PBS, C-mUMSC-EXOs, G-mUMSC-EXOs, and si-circ-G-mUMSC-EXOs and GDM groups (vs. GDM + C-mUMSC-EXOs, ∗P < 0.05, ∗∗P < 0.01, and ∗∗∗P < 0.001). The fasting glucose level of (b) pregnant and (d) fetal mice was measured by blood glucose test strips in control and GDM mice with PBS, C-mUMSC-EXOs, G-mUMSC-EXOs, and si-circ-G-mUMSC-EXOs and GDM groups. ∗∗∗P < 0.001 vs. Control. Figure S2: the relative expression of four candidate miRNAs following different treatment. RT-qPCR assay was performed to detect the level of (a) hsa-miR-338-3p (∗P < 0.05, ∗∗∗P < 0.001 vs. Control; ^^P < 0.01 G-hUMSC-EXOs vs. si-circ-G-hUMSC-EXOs; ##P < 0.01, siNC-G-hUMSC-EXOs vs. si-circ-G-hUMSC-EXOs), (b) hsa-miR-142-3p (∗∗∗∗P < 0.0001 vs. Control; ^^^^P < 0.0001 G-hUMSC-EXOs vs. si-circ-G-hUMSC-EXOs; ####P < 0.0001, siNC-G-hUMSC-EXOs vs. si-circ-G-hUMSC-EXOs), (c) hsa-miR-369-5p (∗∗∗∗P < 0.0001, vs. Control; ^^^^P < 0.0001, G-hUMSC-EXOs vs. si-circ-G-hUMSC-EXOs; ####P < 0.0001, siNC-G-hUMSC-EXOs vs. si-circ-G-hUMSC-EXOs) and (d) hsa-miR-370-3p (∗∗, P < 0.01, ∗∗∗P < 0.001, vs. Control; ^^P < 0.01 G-hUMSC-EXOs vs. si-circ-G-hUMSC-EXOs; ##P < 0.01, siNC-G-hUMSC-EXOs vs. si-circ-G-hUMSC-EXOs) with PBS, G-hUMSC-EXOs, siNC-G-hUMSC-EXOs and si-circ-G-hUMSC-EXOs in L-02 cells. (e) RT-qPCR assay was performed to determine the effects of hsa-miR-338-3p inhibitor on the expression of PTEN (∗∗P < 0.01, ∗∗∗∗P < 0.0001 vs. Control; ^^^^P < 0.0001, vs. siNC + G-hUMSC-EXOs; ##P < 0.01, si-circ + G-hUMSC-EXOs vs. si-circ + G-hUMSC-EXOs + inhibitor miR; &&P < 0.01, si-circ + G-hUMSC-EXOs vs. si-circ + G-hUMSC-EXOs + inhibitor miR). Figure S3 Exosomal hsa_circ_0046060 of hUMSC regulated glucose homeo [file 9218113.f1.zip › 9218113.f1/Supplementary Figures (2).docx]

**Figure S1**


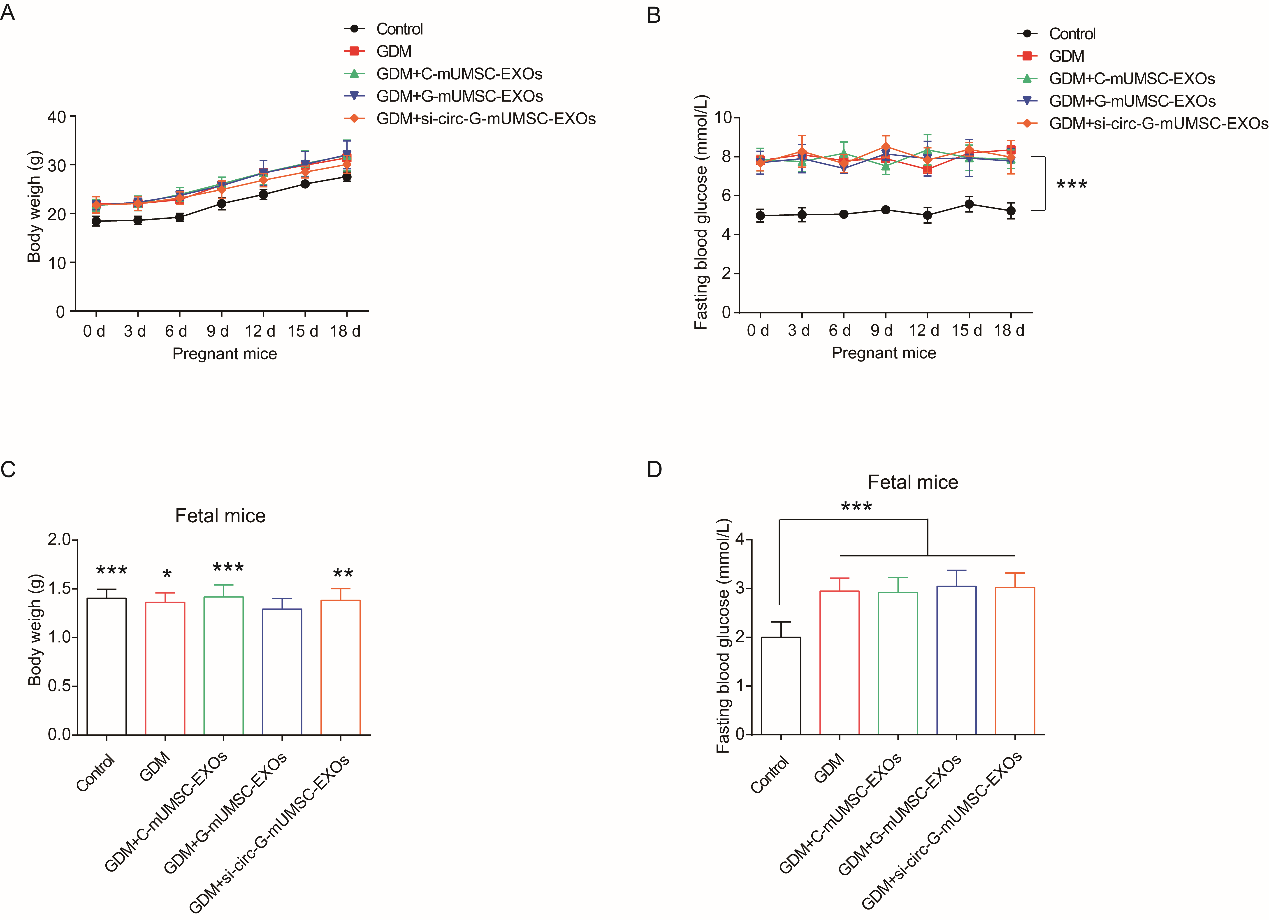


**Figure S1** Basic physiological characteristic of body weight and fasting blood glucose of pregnant mice and fetal mice. The body weight of (A) pregnant and (C) fetal mice was assessed by an electronic weigher in control and GDM mice with PBS, C-mUMSC-EXOs, G-mUMSC-EXOs and si-circ-G-mUMSC-EXOs and GDM groups (vs. GDM + C-mUMSC-EXOs, **P* < 0.05, ***P* < 0.01, ****P* < 0.001). The fasting glucose level of (B) pregnant and (D) fetal mice was measured by blood glucose test strips in control and GDM mice with PBS, C-mUMSC-EXOs, G-mUMSC-EXOs and si-circ-G-mUMSC-EXOs and GDM groups. ****P* < 0.001, vs. Control.

**Figure S2**


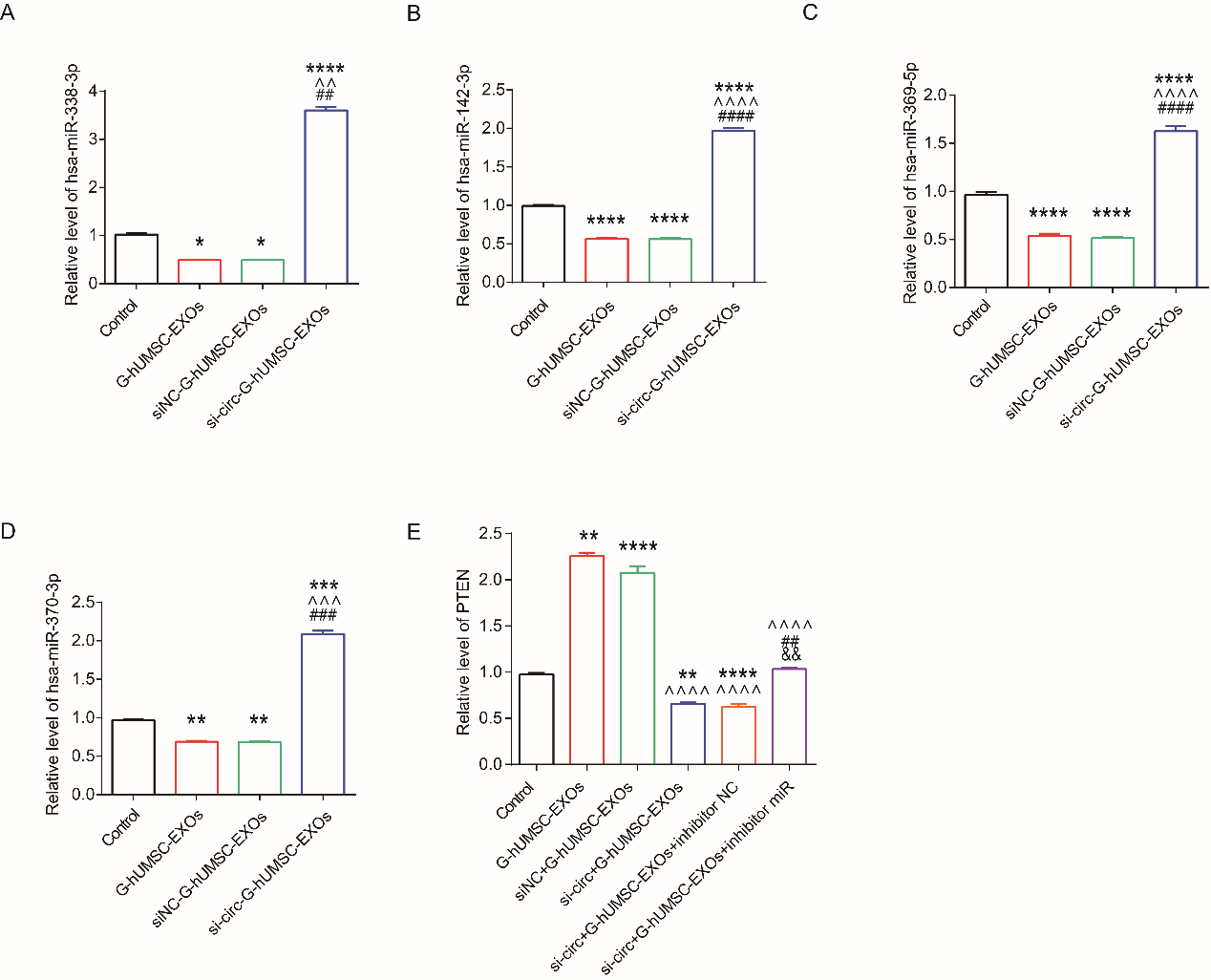


**Figure S2** The relative expression of four candidate miRNAs following different treatment. RT-qPCR assay was performed to detect the level of (A) hsa-miR-338-3p (**P* < 0.05, ****P* < 0.001, vs. Control; ^^*P* < 0.01 G-hUMSC-EXOs vs. si-circ-G-hUMSC-EXOs; ^##^*P* < 0.01, siNC-G-hUMSC-EXOs vs. si-circ-G-hUMSC-EXOs), (B) hsa-miR-142-3p (*****P* < 0.0001, vs. Control; ^^^^*P* < 0.0001 G-hUMSC-EXOs vs. si-circ-G-hUMSC-EXOs; ^####^*P* < 0.0001, siNC-G-hUMSC-EXOs vs. si-circ-G-hUMSC-EXOs), (C) hsa-miR-369-5p (*****P* < 0.0001, vs. Control; ^^^^*P* < 0.0001, G-hUMSC-EXOs vs. si-circ-G-hUMSC-EXOs; ^####^*P* < 0.0001, siNC-G-hUMSC-EXOs vs. si-circ-G-hUMSC-EXOs) and (D) hsa-miR-370-3p (***P* < 0.01, ****P* < 0.001, vs. Control; ^^*P* < 0.01 G-hUMSC-EXOs vs. si-circ-G-hUMSC-EXOs; ^##^*P* < 0.01, siNC-G-hUMSC-EXOs vs. si-circ-G-hUMSC-EXOs) with PBS, G-hUMSC-EXOs, siNC-G-hUMSC-EXOs and si-circ-G-hUMSC-EXOs in L-02 cells. (E) RT-qPCR assay was performed to determine the effects of hsa-miR-338-3p inhibitor on the expression of PTEN (***P* < 0.01, *****P* < 0.0001 vs. Control; ^^^^*P* < 0.0001, vs. siNC + G-hUMSC-EXOs; ^##^*P* < 0.01, si-circ + G-hUMSC-EXOs vs. si-circ + G-hUMSC-EXOs + inhibitor miR; ^&&^*P* < 0.01, si-circ + G-hUMSC-EXOs vs. si-circ + G-hUMSC-EXOs + inhibitor miR).

**Figure S3**


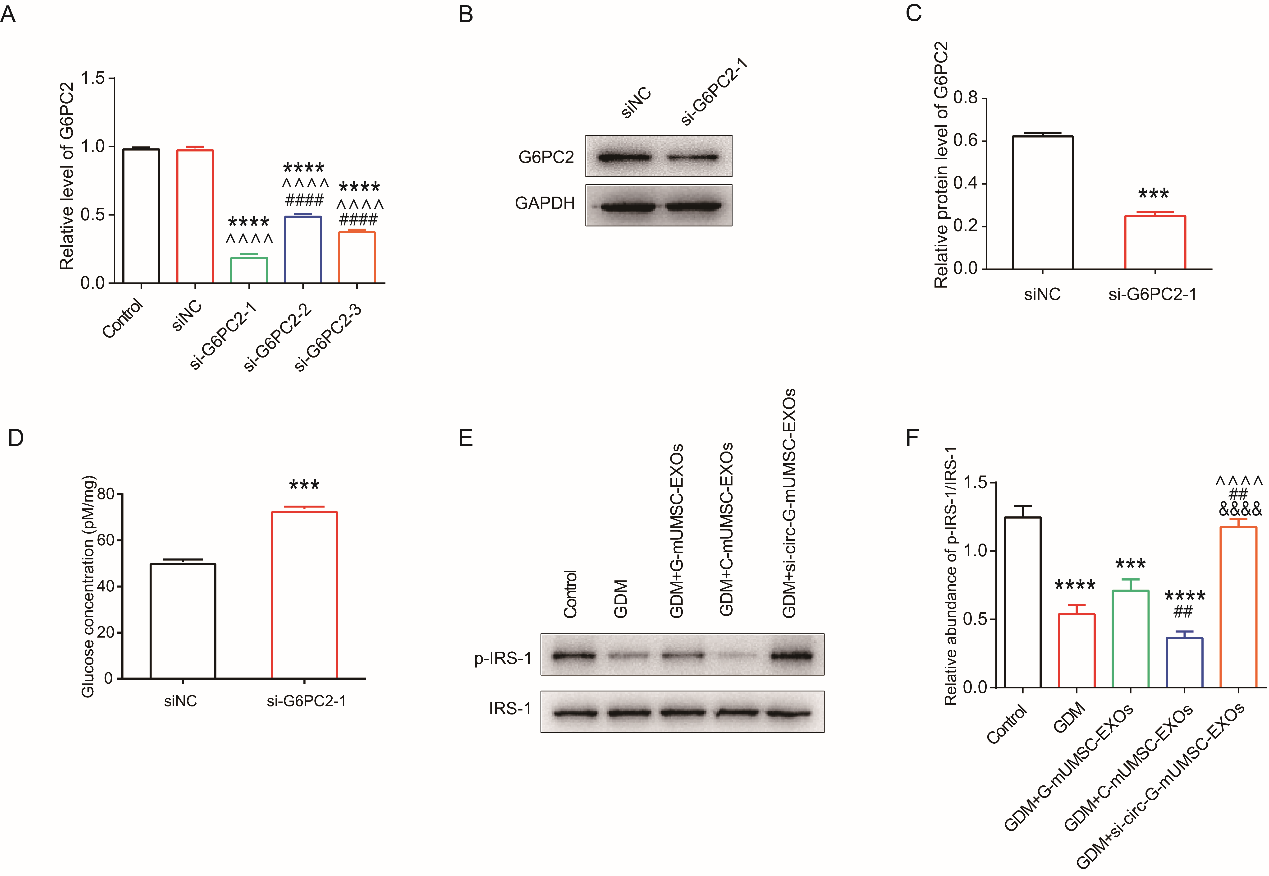


**Figure S3** Exosomal hsa_circ_0046060 of hUMSC regulated glucose homeostasis and induce insulin resistance via hsa-miR-338-3p *in vitro* and *in vivo*. (A) RT-qPCR assay determined the inhibitory effects of three potential siRNAs targeting G6PC2 (*****P* < 0.0001, vs. Control; ^^^^*P* < 0.0001, vs. siNC; ^####^*P* < 0.0001, vs. si-G6PC2-1). (B, C) The protein expression of G6PC2 in L-02 cells by incubation of si-G6PC2-1 was detected by Western blot. ****P* < 0.001 (D) The glucose concentration was measured in siNC and si-G6CP2-1 groups, respectively. ****P* < 0.001 (E, F) Expression of p-IRS-1 and IRS-1 in the L-02 cells was detected by Western blot in control, GDM mice administrated with PBS, C-mUMSC-EXOs, G-mUMSC-EXOs and si-circ-G-mUMSC-EXOs (****P* < 0.001, ****P* < 0.0001, vs. Control; ^^^^*P* < 0.0001, vs. GDM; ^##^*P* < 0.01, vs. GDM+G-mUMSC-EXOs; ^&&&&^*P* < 0.0001, vs. GDM+C-mUMSC-EXOs).
